# Developing healthy eating promotion mass media campaigns: A qualitative study

**DOI:** 10.3389/fpubh.2022.931116

**Published:** 2022-07-29

**Authors:** Carolina Capitão, Raquel Martins, Rodrigo Feteira-Santos, Ana Virgolino, Pedro Graça, Maria João Gregório, Osvaldo Santos

**Affiliations:** ^1^Environmental Health Behaviour Lab, Instituto de Saúde Ambiental, Faculdade de Medicina, Universidade de Lisboa, Lisbon, Portugal; ^2^Laboratório Associado TERRA, Faculdade de Medicina, Universidade de Lisboa, Lisbon, Portugal; ^3^Área Disciplinar Autónoma de Bioestatística, Faculdade de Medicina, Universidade de Lisboa, Lisbon, Portugal; ^4^Faculdade de Ciências da Nutrição e Alimentação, Universidade do Porto, Oporto, Portugal; ^5^Programa Nacional para a Promoção da Alimentação Saudável, Direção-Geral da Saúde, Lisbon, Portugal; ^6^Unidade de Investigação em Epidemiologia (EPIUnit), Instituto de Saúde Pública, Universidade do Porto, Oporto, Portugal; ^7^Laboratório para a Investigação Integrativa e Translacional em Saúde Populacional (ITR), Universidade do Porto, Oporto, Portugal; ^8^Unbreakable Idea Research, Cadaval, Portugal

**Keywords:** health promotion, feeding behavior, mass media campaigns, public health, qualitative research

## Abstract

**Background:**

Involving consumers in the development and assessment of mass media campaigns has been advocated, though research is still lacking. This study aimed to explore opinions and attitudes of citizens, health professionals, communication professionals, and digital influencers regarding the development and implementation of healthy eating promotion mass media campaigns.

**Methods:**

We conducted five semi-structured focus groups, where participants were exposed to the first nationwide mass media campaign promoting healthy eating in Portugal. Through criteria-based purposive sampling, 19 citizens, five health professionals, two communication professionals, and four digital influencers were included. Transcripts were analyzed using Charmaz's line-to-line open coding process.

**Results:**

Main identified themes were: considerations about informative-centered campaigns, health/nutritional issues to address, campaign formulation, target audiences, dissemination channels, and influencers' involvement. Participants favored campaigns focused on practical, transformative, and useful information with simple, innovative, activating, and exciting messages instead of strictly informative campaigns. Health and communication professionals mentioned the importance of adapting the message and dissemination channels to the target audience, addressing the most vulnerable and hard-to-reach individuals, and highlighted the importance of short video format.

**Conclusions:**

Active involvement of the health promotion target audience is crucial for the development and effectiveness of health campaigns. Campaigns need to convey health messages on simple though exciting communication materials, targeted to the most vulnerable subgroups, including deprived, less educated, younger, and older generations.

## Introduction

Dietary risk factors, including low consumption of fruits, vegetables, and legumes, and high consumption of red and processed meats, sugar, and salt, are associated with the development of non-communicable diseases, increasing disability-adjusted life years (DALYs) and mortality. In Portugal, unhealthy eating behaviors were responsible for 7.3% of total DALYs and 11.4% of total deaths in 2019 (1).

Due to the harmful effects of unbalanced diets, improving dietary habits is a clinical and policy priority, at both individual and community levels. Individual-level and health care system-based behavioral change interventions are partly effective (2, 3). However, policy changes at organizational, community, and government levels (in addition to individual-based initiatives) can have a broader, more equitable, and sustainable impact (4). Population-based strategies for healthy eating promotion embrace a media and education domain, including mass media campaigns (4).

Mass media campaigns are being used to increase awareness about different health issues, such as smoking cessation (5), physical activity (6), and healthy eating (7, 8). These interventions may even influence behavior change, as campaigns promoting the use of healthcare services and lifestyle change have been effective in influencing the use of health care interventions (9). Indeed, the World Health Organization recommends campaigns about healthy diets as a strategy to prevent and control non-communicable diseases (10).

In general, to increase the relevance and quality of health care and research, “consumers” involvement is strongly recommended (11, 12). However, little attention has been paid to the potential contributions from diverse groups of stakeholders, such as citizens and health professionals, to the development and evaluation of health campaigns (13), especially regarding healthy eating promotion campaigns. Thus, this study aimed to explore opinions and attitudes of citizens, health professionals, communication professionals, and digital influencers regarding the development of healthy eating promotion mass media campaigns.

## Methods

### Design

This study followed a qualitative design. Data were collected by semi-structured focus groups (FG). Five FG were conducted: three with citizens, one with health professionals and health communication professionals, and one with digital influencers. The FG aimed to understand the perceptions about health promotion campaigns and how to develop effective healthy eating promotion mass media campaigns while discussing the specific case of the “*Eat Better”* campaign, developed by the Portuguese Directorate General of Health.

The COnsolidated criteria for REporting Qualitative research (COREQ) guidelines were followed (14).

### The “*Eat Better*” campaign

The “*Eat Better”* campaign (15) was the first governmental initiative promoting healthy eating at the national level, created and implemented by the National Programme for the Promotion of Healthy Eating, of the Portuguese Directorate-General of Health. Dissemination occurred between November and December 2019. The development, dissemination, and evaluation of the campaign were completely independent, financed by public funds, without the involvement of the food industry and conflicts of interest. The target audience was people living in Portugal aged between 18 and 65 years, with special attention to younger adults as they are at higher risk for low consumption of fruit, vegetables, and legumes, and high consumption of sugary drinks. It was a multi-platform initiative, disseminated on television, radio, social media (through digital influencers), advertising outdoors, public transport, and regional media.

The general aims of the campaign were to: (a) raise awareness of the importance of healthy eating, focusing on health gains from small changes in diet, (b) increase motivation for healthy eating, by demonstrating how easy is to implement small eating changes, and (c) increase competence, by describing how the nutritional recommendations can be achieved, focusing on water, fruits, vegetables, and legumes.

### Participants' inclusion criteria and recruitment

A purposive, criteria-based sampling approach was used. The sampling frame included key factors thought to influence people's perceptions and enhance group dynamics; for the FG with citizens, inclusion criteria (aiming heterogeneity) were: gender, age (between 16 to 34 and 54+ years), educational level (higher or no higher education), living in different regions of Portugal (rural and urban settings), and having being exposed or not to the “*Eat Better”* campaign; for the FG with professionals: academic background and professional activity (medical doctors, nutritionists, psychologists, nurses, and professionals working in health communication and mass media campaigns); and for the FG with digital influencers: digital platform presence, health domain (nursing, nutrition), and having or not been involved in the “*Eat Better”* campaign.

For the recruitment process, invitations by e-mail were sent to members of different organized groups of citizens, individuals who had already participated in other FG (for different research projects), and acquaintances to members of the research team. Regarding digital influencers, potential participants (content creators with ≥20 thousand followers, addressing topics related to healthy eating, physical activity, health, and lifestyle) were identified by searching on different social media platforms and were contacted *via* e-mail. The e-mail contacts included the informed consent form with general information about the topic of the FG (not providing the FG script).

### Focus groups description

The five FG were carried out between February and April 2020. Two FG were conducted at the Lisbon School of Medicine campus (with citizens), two at the Portuguese Directorate-General of Health headquarters (with professionals and digital influencers), and one online *via* Zoom^®^ platform (with citizens) due to restrictions related to the COVID-19 pandemic, which allowed participants from different regions of the country to be included. All FG were audio-recorded and the mean discussion time was 92 min.

A total of 30 individuals participated in the FG, including 19 citizens, five health professionals, two communication professionals, and four digital influencers (three nutritionists and one nurse). The number of participants by FG varied between four and seven. The characteristics of citizens are described in Table 1 and of health and communication professionals and digital influencers in Table 2.

**Table 1:** Characteristics of the study participants included in the focus groups with citizens conducted to explore opinions and attitudes regarding the development of healthy eating promotion mass media campaigns.

**Focus group**	***n***	**Age (years), median (range)**	**School years completed, mean**	**Female, %**	**Setting, location**
Citizens 1	6	47 (21–63)	17.8	50.0	Face-to-face, Lisbon
Citizens 2	6	31 (16–53)	14.7	50.0	Face-to-face, Lisbon
Citizens 3	7	37 (21–65)	14.3	28.6	Video conference

**Table 2:** Characteristics of the study participants included in the focus groups with health and communication professionals and digital influencers conducted to explore opinions and attitudes regarding the development of healthy eating promotion mass media campaigns.

**Focus Group**	***n***	**Profession**	**Participant ID**	**Female, %**	**Setting, location**
Professionals	1	Medical doctor	A	57.1	Face-to-face, Lisbon
	1	Biologist specialized in science communication	B		
	1	Specialist in strategic communication applied to science, culture, and art	C		
	2	Nurses	D, G		
	2	Nutritionists	E, F		
Influencers	3	Nutritionists	A, C, D	100.0	Face-to-face, Lisbon
	1	Nurse	B		

The FG were conducted by a moderator, a male health psychologist (MSc) with extensive training in qualitative research, together with a co-moderator (male nutritionist or female health psychologist, varying with the FG). A note-taker (female nutritionists) was always present in the room to capture the main themes discussed, as well as the non-verbal behaviors of the participants. The FG started with a brief presentation of the FG structure and aims. The moderator and co-moderator conducted the FGs according to semi-structured scripts (which differed according to the FG participants and considered previous FG emergent items and dimensions). The final FG scripts can be found in Supplementary Figure S1. The moderator disclosed that any of the elements of the research team involved in the data collection and posterior content analysis were neither involved in the development nor the implementation of the “*Eat Better”* campaign. In each FG, only the participants, the moderator, the co-moderator, and the note taker were present in the room.

To introduce the discussion regarding the “*Eat Better*” campaign, participants were exposed to the promotional materials (video, poster, and social media posts) about 20–30 min after the beginning of the FG.

### Data analysis

Audio recordings were fully transcribed by an experienced researcher and writer (with extensive experience in FG transcriptions), according to a pre-defined transcription set of guidelines. The complete set of transcriptions formed the corpus and was read by two researchers independently to get familiar with and identify the main dimensions emerging from the corpus (intuitive reading). For completing the open coding process, the same authors analyzed the transcripts, line by line, using a constant comparison approach (between units of meaning—each participant occurrence—and the complete corpus: Charmaz's open coding process) (16). After this independent coding, the two researchers reached interpretative consensus (triangulating also with the FG moderator) and built up the code system (available in the Supplementary Material). Subsequently, the codes were grouped into themes and subthemes, according to the similarity between the identified codes (axial coding), using the constant comparison method. The relationships between themes were reviewed, by re-reading the transcripts already analyzed, to compare and develop new dimensions, from a hermeneutic perspective. Finally, the relationships between themes and subthemes were examined to define the central theme of the study and, in turn, to elaborate a conceptual framework (conceptual coding). The codes and themes that emerged were reviewed by the FG moderator and one of the co-moderators, ensuring triangulation in data interpretation. The analysis was performed in MAXQDA, version 18.0.

After content analysis, participants were contacted again and asked to provide additional feedback on the findings for validating meaning and the researchers' analysis and interpretations.

### Human participant compliance statement

The study protocol was reviewed and approved by the Ethical Committee of the Lisbon Academic Medical Centre and the study was conducted according to the principles of the Declaration of Helsinki (17). Before the FG, all participants were informed that the sessions would be recorded for later transcription and that the data analysis would be carried out in a grouped manner, maintaining anonymity. All participants were asked to read and sign informed consent forms. Commuting costs were covered with a 10 gift card delivered to each participant.

## Results

During the FG, health mass media campaigns and healthy eating promotion campaigns were discussed. The main themes derived from the data are described below. Table 3 shows some of the supporting *verbatim* related to each main theme and the full list is presented in Supplementary Table S2.

**Table 3:** Supporting verbatim exemplifying the six main themes emerging from the narratives of the focus groups conducted with citizens, health and communication professionals, and digital influencers to explore opinions and attitudes regarding the development of healthy eating promotion mass media campaigns.

**Main theme**	**Supporting verbatim**
Informative campaigns	“If we are thinking about informative campaigns... mass media, right?..., if this is where we are focusing on... (…) these actions should be combined with other punctual interventions (…) of more proximity...” [Professionals FG, E; Line/s 252 - 256]
Health/nutritional issues to address	“Making things maybe less…, that people like less…, into something pleasant to the palate and the sight, (…) And to associate with good experiences” [Professionals FG, G; Line/s 1119 - 1122]
Campaign formulation	“What the brand has to say, or the project has to say must be relevant, it has to be exciting, it has to be clear, true, and original. (…) The quality of the insight is measured by these five elements.” [FG professionals, C; Line/s 1134 - 1161] “There's another level that is relevance by identification, which is where the influencers come in… It is the relevance that is like: ≪Eh, if Cristiano Ronaldo wears, I also want to wear≫. This is relevance by identification. And the other is relevance by improvement. That is ≪What you are saying to me will improve my life≫. What you told me today, I will implement it right away [?] at breakfast…” [FG professionals, C; Line/s 1136-1140] “But this is important (…) to simplify the message. This is... I think it is super-important to have an effect. (...) focusing only on one behavior rather than trying to change everything simultaneously.” [Professionals FG, F; Line/s 243 - 247]
Targeted audiences	“Children will be the vehicle for that information, and it focuses on behavioral change that carries the message to families and, in some way, is a source of contagion.” [FG professionals, E; Line/s 115 - 117]
Dissemination channels	“I think that television [to reach less urbanized areas] continues to make a lot of sense.” [Citizens FG3, A; Line/s 842 - 842]
Influencers' involvement	“People identify with us; people see themselves in our place and that is what makes them follow our work. Of course (…), we take this to promote health and well-being... But that's it..., that is why people identify with us, people... ≪I've also been in this position where she's talking... I also do what she does... So, I also want to eat what she eats≫” [Influencers FG, C; Line/s 376 - 381]

*FG, focus group*.

### Considerations about informative-centered campaigns

Some participants perceived most health mass media campaigns as impersonal and uninteresting. Campaigns were recognized, typically, as an ineffective strategy to promote behavioral changes, as they were considered a mere information vehicle, lacking narratives evoking emotions. To overcome this issue, one health professional suggested that campaigns should be complemented with proximity interventions (e.g., initiatives in which health professionals, as well as public figures, from sports or science, would work out the key messages of the campaigns together with citizens). Notwithstanding, nutritional (informative content-based) literacy was understood as an essential element for healthy eating choices, even though personalized information about food habits (suited to target audiences) was deemed essential, due to interindividual differences.

### Health/nutritional issues to address

In all FG, participants identified barriers to healthy eating, such as the unpleasantness associated with some healthy foods and the emotional connection that influences food choices and often compromises the adherence to a healthy food pattern.

Participants suggested that healthy eating campaigns should aim to modify the perception of what is (un)pleasant, for example, by providing recipes that do not sacrifice the taste or pleasure associated with food. In parallel, the focus on emotions and feelings associated with healthy eating behaviors was also proposed as a key point to promote changes.

### Campaign formulation

In the FG with health and communication professionals, it was highlighted that health campaigns need to communicate a central message—a message that creates the need to adopt a different behavior. This message should convey an insight that is based on at least four out of five key elements: relevance of the message (to the target audience), truthiness, simplicity, originality, and excitement.

It was referred that the message can be made relevant by identification or perceived improvement. The first can be present when public figures/influencers are involved in the campaign (which was advocated in all FG). It exists when the audience identifies themselves with the public figures (participating in the campaign) that they consciously or unconsciously want to follow (or even be like). For this to happen successfully, the message should absorb the acclaimed characteristics of the public figure, so that the relationship between both parties is more easily recognized and remembered. The second is present when the target public identifies a clear benefit associated with the transmitted message and recognizes that the promoted behavior can be implemented in their lives.

The insight's key element of truthiness entails interpreting statistics and facts to turn them into compelling and emotional insights that are effective at promoting behavior change, instead of just presenting them.

To clearly transmit the campaign's message, it should be simple to interpret and practical. Whenever possible, campaigns should challenge the public with concrete tasks/behaviors, and eating behavior changes ought to be addressed in a phased manner, to avoid the feeling of being overwhelmed. Campaigns should present new information to increase interest and excitement. The message also needs to be adapted to the target population, which implies a segmentation of the audience according to their values and interests.

When discussing communication formats, the use of storytelling videos conveying individuals was perceived to be the most effective approach.

The communication strategy should aim to create an identity over time, making it recognizable by the audience and perceived as trustworthy, which implies regularity and coherence in the messages transmitted.

### Targeted audiences

Nutritional literacy promotion was believed to be most effective when targeted to younger audiences, when many habits are more easily formed, also considering the intergenerational influence on behavior (from youngsters to adults). Moreover, it was identified the need for healthy eating campaigns to target less urbanized areas, as participants perceived that these regions are underprovided of nutritional literacy programs.

### Dissemination channels

Along with other communication channels (e.g., public transportation, clinic waiting rooms, and university campuses), television was pointed out as an advantageous channel to reach less urbanized areas. However, it was noted that this format is falling into disuse, as the preference and availability for streaming services or internet entertainment are increasing.

### Influencers' involvement

Influencers' (e.g., celebrities, digital influencers) involvement was recognized as a valuable element of a health campaign, because of their wider audience reach. Digital influencers' communication channels (social media platforms) were regarded as highly personal, promoting identification and empathy with the source of the information. Nonetheless, digital influencers were also recognized as responsible for the dissemination of “eating trends”.

The digital influencers participating in the FG revealed that they welcome partnerships with public health promotion campaigns if the message conveyed is aligned with their digital influencer professional project. A preference for active partnerships, where the creator is responsible for the materials, instead of just sharing a preformatted message, was also expressed. This was reinforced as essential to the influencers' involvement success, as it is a key determinant for influencers' followers to ascertain the authenticity of the message.

## Discussion

Our findings contribute to the understanding of how to develop healthy eating promotion mass media campaigns, from the perspective of citizens, health and communication professionals, and digital influencers.

Across the five FG, it was discussed that mass media health campaigns should be complemented with multi-agent proximity-based initiatives, address barriers to healthy eating, and aim to modify the target audience's perceptions through a message based on relevance, excitement, simplicity, truthiness, and originality. Campaigns targeting younger audiences, investing in dissemination channels that target more difficult-to-reach segments of the population, and involving familiar faces, such as digital influencers, were also highlighted.

Information-centered health mass media campaigns were considered an ineffective strategy to change health behavior. Although mass media health campaigns are expected to have small-to-moderate effects on individuals' health knowledge, attitudes, and behaviors, by reaching large proportions of a target population, it could translate into major population impacts (18). This type of public health strategy has been effective in improving dietary intake (19). Nevertheless, success is mainly observed when the campaign is part of a multicomponent program or comprehensive strategy (20), where health professionals play an essential role.

Indeed, health professionals (from different areas and backgrounds) should be part of the development of healthy eating promotion campaigns, following a participatory approach (11). Besides being involved in complementary actions in different contexts, as mentioned (e.g., in schools), health professionals can help define needs and priorities based on their clinical/field experience/background and develop more practical messages and activities to increase the effectiveness of the behavior change promotion (e.g., by developing recipes as suggested in the FG).

As a general consideration, health policies should promote the development of personal values that guide individuals to the healthiest possible decision-making. For this to happen, the promoted health behaviors have to be considered relevant for the short term, which is even more important for young people [who tend to have less capacity for long-term self-regulation (21)]. This can be achieved if campaigns' messages convey relevance, excitement, simplicity, truthiness, and originality, as mentioned by a communication professional in the FG. It was also stated that messages should be formulated in a simple language and should make the audience feel challenged, to improve engagement.

Participants in this study recognized that adherence to a healthy food pattern is challenging. The main barriers identified were the unpleasantness often associated with healthy foods and the emotional connection that influences food choices. Other barriers have been identified, such as the time needed to prepare healthy food, the perceived cost of healthy eating, and social influences (22). When promoting eating behavior change, it was stressed that a stepped approach is necessary and that the health campaign should create an identity over time, which entails regularity and coherence related to the messages transmitted. In fact, broad and limited-duration health campaigns targeting the adoption of multiple behaviors are not thought to be successful (4). The campaign's identity needs to communicate who the campaign promoter is, what it stands for (regarding values and goals), how it works, and the intended relationship with the subject and the audience; it should also include the perceptions people hold about the issue of the campaign (23), which reinforces the need for formative research to adequately segment the target population before campaign development, identifying the most appropriate communication strategies (24).

Amidst other dissemination channels, television was regarded as advantageous, in particular, to reach less urbanized areas. Television has been considered the most effective media channel to reach most people (13) and the most recalled delivery channel in health campaign assessments (7, 25). One of the main reasons is that television content is consumed passively and easily, implying little effort from the audience to receive the message (13). Additionally, healthy eating has been negatively linked to watching television, meaning that it could effectively reach audiences in need of campaigns promoting healthy eating (26). Regarding difficult-to-reach population strata, and in line with participants' opinions, television is effective in reaching audiences with basic education and lower income, which could be attributed to higher consumption of television (7). These considerations are relevant when aiming to reduce health inequities. Health inequities are caused by a complex set of interrelated factors at the environmental, societal, socioeconomic, individual, and behavioral levels (27). The causes include, but are not limited to, differential exposure to environmental health agents (27) and deliberate efforts to reach underserved populations, namely when defining mass media campaigns dissemination, are necessary.

Nutritional literacy promotion was believed to be more effective when targeted to younger audiences. It has been recognized by health professionals that social media provides a pivotal opportunity to reach and engage with young adults that may not otherwise seek out health professionals in more traditional settings (28). However, despite its growing popularity, public and non-profit sectors are underusing social media in a way that maximizes their capacity to engage with their followers (29). Social media can act as a platform to deliver and increase exposure to evidence-based key messages of health promotion campaigns and to encourage young adults to participate and engage (30). Young adults are interested in using social media for learning about nutrition-related information and recognize the usefulness of social media channels to learn about recipes and healthy eating (30). In our study, digital influencers were available to be involved in health promotion campaigns if the messages are aligned and tailored to narratives fitting their professional project.

In the present study, we sought out perspectives from different backgrounds and professional occupations that could influence the way that participants would look at the subject under study. Although this study was conducted with a diversified sample of Portuguese participants, the collected perspectives are surely not limited to Portuguese reality. As discussed, many opinions expressed in the FG agree with current literature, highlighting priorities that should be considered when dealing with communities, even outside Portugal. Nevertheless, data saturation may have not been reached due to the relatively small number of FG per type of participant, mainly with health and communication professionals and digital influencers. Additional research is needed to clarify and expand the obtained results. Nonetheless, these findings contribute to the overall knowledge in this field of research and provide valuable and useful information for informing the development of health campaigns. Despite efforts to create a comfortable environment, some opinions and attitudes perceived as rare or unpopular may not have been shared and participants may have expressed the opinions that they thought the researchers wanted to hear and future studies would benefit from a data collection methods triangulation (namely, with individual in-depth interviews).

## Conclusion

The involvement of citizens, health and communication professionals, and digital influencers allowed us to understand different and complementary perspectives on how healthy eating promotion campaigns should be developed, regarding goals, messages' content and formulation, means of dissemination, and the usage of social media platforms. Our findings provide relevant insights that should be considered when developing health mass media campaigns. Actively involving the public at the different stages of public health campaigns can be a relevant determinant of success.

## Data availability statement

The raw data supporting the conclusions of this article will be made available by the authors, without undue reservation.

## Ethics statement

The studies involving human participants were reviewed and approved by Ethical Committee of the Lisbon Academic Medical Centre. The patients/participants provided their written informed consent to participate in this study.

## Author contributions

OS, RF-S, and AV designed the study and moderated the focus groups. CC and RM analyzed the data and wrote the first draft. All authors reviewed and made valuable contributions to the manuscript.

## Funding

This work was supported by the Portuguese Directorate-General of Health. The Directorate-General of Health had no role in the design, the analysis of this work, and the decision to publish. Researchers affiliated with the Portuguese Directorate-General of Health reviewed and made valuable contributions to the manuscript. The writing of the manuscript was also supported by funds from Fundação para a Ciência e a Tecnologia to ISAMB (ref. UIDB/04295/2020 and UIDP/04295/2020).

## Conflict of interest

The authors declare that the research was conducted in the absence of any commercial or financial relationships that could be construed as a potential conflict of interest.

## Publisher's note

All claims expressed in this article are solely those of the authors and do not necessarily represent those of their affiliated organizations, or those of the publisher, the editors and the reviewers. Any product that may be evaluated in this article, or claim that may be made by its manufacturer, is not guaranteed or endorsed by the publisher.
