# Supplementary material for: Developing healthy eating promotion mass media campaigns: A qualitative study
Source: Front Public Health. 2022 Jul 29;10:931116. doi: 10.3389/fpubh.2022.931116 (PMC9372615; doi:10.3389/fpubh.2022.931116)
Supplement: Supplementary file 1 [file Data_Sheet_1.docx]

Developing healthy eating promotion mass media campaigns: a qualitative study

**Supplementary material**

[Supplementary Figure 1. Diagram illustrating the discussion-flow script for the Focus Group moderation 2](#_Toc101523459)

[Supplementary Table 1. Supporting verbatim exemplifying the six main categories emerging from the narratives of the focus groups conducted with citizens, health and communication professionals, and digital influencers to explore opinions and attitudes regarding the development of healthy eating promotion mass media campaigns 3](#_Toc101523460)

[CODE SYSTEM 7](#_Toc101523461)

**Introduction:** Presentation, warm-up, and semantic association exercise.

⇓

| **Citizens**  **Information sources:**   - Usual information sources - Most used sources about nutrition - Most credible sources |  | **Professionals**  **Communication strategies with users/population:**   - Main messages disseminated among users/population about food/nutrition - Sources of information for the construction of disseminated content - Strategies to tailor messages to the audience |  | **Digital Influencers**  **Communication strategies:**   - Main messages posted on social media - Contents/themes that generate more interest/interaction - Sources of information for the construction of promoted content - Criteria for agreeing to promote third-party content - Strategies to tailor messages to the audience |
| --- | --- | --- | --- | --- |
| ⇓ |  |  |  |  |
| **Exposure to information campaigns:**   - Healthy eating promotion campaigns remembered - Characteristics remembered (and what makes it rememberable) - Opinions on those campaigns |  |  |  |  |

⇓

**“Eat Better” campaign:**

- Awareness: contact with campaign materials & through which means
- Implicit/explicit campaign messages
- Adequacy of the means to transmit the key messages (positive/negative aspects)
- Possible impacts of the campaign (target population reach; changes in attitudes/behaviour of the target population): for the Portuguese & the target population

⇓

**Benchmarking for behavioural change promotion:**

- If you were invited by the Ministry of Health to design a campaign to promote healthy eating, what recommendations would you make?

# Supplementary Figure 1. Diagram illustrating the discussion-flow script for the Focus Group moderation

# Supplementary Table 1. Supporting verbatim exemplifying the six main categories emerging from the narratives of the focus groups conducted with citizens, health and communication professionals, and digital influencers to explore opinions and attitudes regarding the development of healthy eating promotion mass media campaigns

| Main category | Supporting verbatim |
| --- | --- |
| Informative campaigns | “Here the campaigns are not very personal. (…) They are impersonal.” [Influencers FG, B; Line/s 670 - 672]  “all the campaigns..., Pap smears, vaccinations, (…) in the waiting room, fixed with a very cute poster. Is it not? What... People don't... they can read it but it doesn’t touch them.” [Influencers FG, B; Line/s 647 - 650]  “if we are thinking about informative campaigns... mass media, right?..., if this is where we are focusing on... And it is inseparable, I would say, to achieve more results, these actions should be combined with other punctual interventions and more ..., of more proximity... That is, I think it can be an addition ...” [Professionals FG, E; Line/s 252 - 256]  “We are more and more aware of what we lack in terms of vitamins, minerals, and everything else... And knowing that this food, which we may not normally include in our diet, has certain vitamin or mineral complexes..., or the energy is X, but it does not have too much fat, we can make the right choice. I think that nowadays this type of information is very important.” [Citizens FG1, A; Line/s 965 - 969]  “Yes, the problem is this. The problem is to know what information is right. For me... (...) For someone else, it may be other information... Because we are not all the same, which is normal...” [Citizens FG1, C; Line/s 209 - 212] |
| Health/ nutritional issues to address | “how do we discuss with the population and get them to create certain habits if everything we are saying is anything but appealing” [Professionals FG, G; Line/s 422 - 423]  “The emotional connection to food goes beyond the issue of being seen as a therapy.” [Professionals FG, F; Line 429]  "Making things maybe less…, that people like less…, into something pleasant to the palate and the sight, (…) And to associate with good experiences” [Professionals FG, G; Line/s 1119 - 1122]  “There is a missing of ..., examples. Because ..., I don't know ... Not all of us can spend two hours in a kitchen ..., and sometimes we eat worse ... I try to go there for the taste, whatever ... Maybe ... «Look, here is a recipe that you can make in thirty minutes that is very healthy and even tasty ...»” [Citizens FG2, C; Line/s 1240-1243]  “we are not selling vegetables (in quotation marks). We are trying to sell how vegetables are going to make you feel. That is, we do not want to tell people something along the lines: «Eat beans because it has x vitamins and minerals.» Because that enters at a hundred, a little bit..., and leaves at two hundred. We may want to say: «Eat beans because it can make you stronger», etc. (...) It is not showing what it is, (…) but what is going to make people feel. (…) we don’t buy a car because we want to have that car. We buy a car because we’re going to feel powerful inside that car.” [Influencers FG, A; Line/s 761 - 773] |
| Campaign formulation | “This is called «insight»”. (…) Which is usually based on the truth, or even is inspired..., is often inspired by a truth…” [FG professionals, C; Line/s 529 - 537]  “What the brand has to say, or the project has to say has to be relevant, it has to be exciting, it has to be clear, true, and original. (…) The quality of the insight is measured by these five elements.” [FG professionals, C; Line/s 1134 - 1161]  “But now this is important, which is... to simplify the message. This is... I think it is super-important to have an effect. (...) focusing only on one behavior rather than trying to change everything simultaneously.” [Professionals FG, F; Line/s 243 - 247]  “Why does it work [showing recipes]? Because it tells people what to do. (…) It is practical.” [Influencers FG, B; Line/s 1482 - 1483]  “Sometimes the challenge is missing..., in a campaign...” [Citizens FG2, B; Line 851]  “the consumer…, the people outside turn…, they turn their heads when there is something new. (…) there are three thousand messages or symbols of communication per day, and if it is not something that is a little bit out of the ordinary, we are in trouble.” [FG professionals, C; Line/s 781 - 788]  “There are already studies that show that the video is the new…, it is the new form of content that people will identify with more ...” [Influencers FG, A; Line/s 291 – 292]  *“There are no people [in the campaign] and the fact that the Directorate is the General Directorate of Health gives a..., that is ... It gives credibility... That is, I think it is important, but it could have a face... (...) And... familiar faces, not those faces of extra people, beautiful to... die…”* [Influencers FG, B; Line/s 1364 - 1368]  “Identity is part of lasting communication. It's like… a body. Now, what he says varies. Therefore, advertising is not design. Design is lasting, (…) this communication is a communication that is…, he’s going to say this… but maybe next year he has to say something else. Even if only to surprise. It’s like Compal^®^ does. People believe when they see the identity of that brand. It’s a trusted brand.” [Professionals FG, C; Line/s 1024 - 1030] |
| Targeted audiences | “It made sense there because it was the children... (…) There is even the expression that is used to say... that an old donkey does not learn languages. Therefore, we must start with younger guys...” [FG professionals, C; Line/s 929 - 933]  “children will be the vehicle for that information and it focuses on behavioral change that carries the message to families and, in some way, is a source of contagion.” [FG professionals, E; Line/s 115 - 117]  “But around [my village] I don't see great communication about nutrition, about food..., I don't see a great..., say..., a great campaign of... which is an important thing, of course, isn't it?” [FG3 citizens, G; Line/s 431 - 434] |
| Dissemination channels | “I think that television, for this [to reach less urbanized areas], continues to make a lot of sense.” [Citizens FG3, A; Line/s 842 - 842]  “people watch less television. (…) They consume fewer television advertisements... Go to Netflix, go to HBO. And so..., so... I don't watch television...” [Influencers FG, C; Line/s 982 - 985] |
| Influencers’ involvement | “It makes perfect sense for them [influencers] to be involved. (…) Because I think they reach more people. It's as simple as that.” [Influencers FG, A; Line/s 735 - 737]  “people identify with us, people see themselves in our place and that is what makes them follow our work. Of course, afterward, we take this to promote health and well-being ... But that’s it ..., that is why people identify with us, people ... «I’ve also been in this position where she’s talking... I also do what she does ... So, I also want to eat what she eats, I also want to support her ...»” [Influencers FG, C; Line/s 376 - 381]  “I also believe that influencers and YouTubers... I remember (...) a YouTuber, who was a vegetarian, came after saying that "Attention, it is not because I am a vegetarian that you have to be..." because there were a lot of people who stopped eating meat without going to a nutritionist …” [Citizens FG2, A; Line/s 409 - 413]  “I have accepted some [partnerships] that did not make sense to the project but also ..., now I wouldn't accept them... (...) even if it was well paid, I wouldn't ..., I wouldn't accept it... but it has to make sense to the project, I think...” [Influencers FG, D; Line/s 479 - 485]  “I think ..., [it went well] because you were real. Each of you did it in your way, each one shared in their way, there was no one formatted thing that everyone... Yes, they were all talking about the same ... [?], It was a campaign ... (...) But each one did it in their own ..., their recipe, their personal signature ... And I think it went well because of that.” [Influencers FG, C; Line/s 1230 - 1235] |

FG, focus group.

# CODE SYSTEM

**1. Semantic association of words/ concepts related to food**

1.1 Being healthier

1.2 Healthy eating

1.3 Eating legumes

1.4 Eating fruits and vegetables

1.5 Drinking water

**2. Information search flow**

2.1 Information sources about food

2.1.1 Personal experience and common sense

2.1.2 Knowledge of others

2.1.3 Health professionals

2.1.4 Technical sources

2.1.5 Internet

2.1.6 Food campaigns

2.1.7 Traditional media

2.1.8 Does not search information about nutrition

2.2 Information’s availability

2.3 Cognitive processing of information

2.3.1 Strategies to filter or to clarify information

2.3.1.1 Based on personal experiences or habits

2.3.1.2 Consulting others

2.3.1.3 Consulting health professionals

2.3.1.4 Consulting the internet

2.4 Strategies to assess information’s credibility

2.4.1 Based on the information sources’ credibility

2.4.1.1 Reliable sources

2.4.1.2 Unreliable sources

2.4.2 Web Pages that assess the papers’ veracity

2.4.3 According to the frequency of information available

2.4.4 According to preconceived beliefs

2.4.5 According to others’ experiences

**3. Perceptions about food and nutrition**

3.1 Importance of healthy eating

3.2 Knowledge about nutrition

3.3 Characteristics of healthy eating

3.4 Palatability in conflict with health

3.5 Nutritional quality of food donations

3.6 Food taxes/ fees

**4. Eating behaviours**

4.1 Behaviours and generations

4.2 Determinants of food choices

4.3 Changing habits and behaviours

4.3.1 Information and behaviours

4.3.2 Changing eating behaviours is difficult

4.3.3 Changing eating behaviours according to trends

4.3.4 Characteristics of the behaviour change approach

4.3.5 Behaviour change facilitators

4.3.6 Factors of resistance to change

4.3.7 Difficulty in maintaining behaviours

4.4 Influence of self on others

**5. Health campaigns**

5.1 Confusion between campaigns and advertisements

5.2 Recalled campaigns

5.2.1 Health care campaigns

5.2.2 Food campaigns

5.2.3 Physical activity promotion campaigns

5.2.4 Anti-smoking campaigns

5.2.5 Road safety campaigns

5.2.6 Drowning prevention campaigns

5.2.7 Recycling campaigns

5.3 Recognized general characteristics

5.4 Considerations for campaign development

5.4.1 Problem identification

5.4.2 Ensuring access to the necessary resources

5.4.3 Necessary to plan active communication

5.4.4 Message source

5.4.5 Necessary to complement information campaigns

5.5 Campaign goals

5.6 Targeted audience

5.7 Message content

5.7.1 Simple and practical messages

5.7.2 Novelty

5.7.3 Messages that promote reflection

5.7.4 Shocking messages

5.7.5 Funny messages

5.7.6 Message focus

5.8 Message transmission

5.8.1 Language to use

5.8.2 Graphic design

5.9 Approaching healthy eating through campaigns

5.9.1 Guidelines

5.9.2 Message focus

5.9.3 Difficulties

5.9.4 Contents

5.10 Campaign or information dissemination

5.10.1 Frequency and promotion of repeated exposure

5.10.2 Choosing information channels and dissemination strategies

5.10.3 Food campaign timings

5.10.4 Channels and means of dissemination

5.10.4.1 Traditional dissemination channels

5.10.4.2 Internet

5.10.4.3 Dissemination through influencers

5.10.4.4 Physical locations

5.11 Evaluating campaigns

5.12 Characteristics that influence campaigns effectiveness

5.12.1 Depends on the target audience

5.13 Campaigns targeting children

5.13.1 Campaigns in schools

5.13.2 Promotion of healthy eating through television fiction

5.13.3 Using catching elements to capture children's attention

**6. “Eat better, a recipe for life” campaign**

6.1 Knowledge and considerations about the Portuguese Directorate-General for Health

6.2 Campaign recognition

6.3 Target audience

6.4 Goals

6.5 Messages interpretation

6.5.1 General message

6.5.2 Promoted eating habits

6.5.3 "Drink more water (but without sugar)"

6.5.4 "Starting the day"

6.5.5 "Step by step"

6.5.6 "Adding new friends"

6.6 Positive opinions

6.6.1 General

6.6.2 Message

6.6.3 Design

6.7 Negative opinions

6.7.1 General

6.7.2 Message

6.7.3 Design

6.7.4 Video

**7. Social media**

7.1 General considerations

7.2 Digital Platforms

7.2.1 Instagram

7.2.2 Facebook

7.2.3 TikTok

7.2.4 Choosing digital platforms

7.3 Influencers

7.4 Transmitting messages through social media

7.4.1 Influencers’ influence

7.4.2 Identification is important for effective message transmission

7.5 Contents

7.6 Objectives for social media usage

7.7 Partnerships

7.7.1 Partnership management

7.7.1.1 Criteria for influencers’ acceptance

7.7.1.2 Criteria for influencers’ non-acceptance

7.7.2 Guidelines for influencers’ involvement in campaigns

7.7.3 Health entities and social media partnerships

7.8 Indicators of success
